# Clinical and laboratory factors related to acute isolated vertigo or dizziness and cerebral infarction

**DOI:** 10.1002/brb3.1092

**Published:** 2018-08-11

**Authors:** Lian Zuo, Yiqiang Zhan, Feifeng Liu, Chen Chen, Luran Xu, Zeljka Calic, Dennis Cordato, Cecilia Cappelen‐Smith, Yunfeng Hu, Gang Li

**Affiliations:** ^1^ Department of Neurology East Hospital Tongji University School of Medicine Shanghai China; ^2^ Department of Internal Medicine Shanghai Yangsi Hospital Shanghai China; ^3^ Department of Neurophysiology Liverpool Hospital Liverpool NSW Australia; ^4^ Ingham Institute for Applied Medical Research Liverpool NSW Australia; ^5^ South Western Sydney Clinical School University of New South Wales Sydney Australia; ^6^ The George Institute for Global Health University of New South Wales Sydney Australia; ^7^ Department of Neurology Shanxi Provincial People's Hospital Shanxi Province China

**Keywords:** cerebral infarction, dizziness, isolated vertigo, neuron specific enolase, risk factors, vertebral artery abnormalities

## Abstract

**Objective:**

To clarify the relationship of clinical factors with isolated vertigo or dizziness of cerebrovascular origin.

**Methods:**

Clinical data of patients admitted in East Hospital from Jan. 2015 to Apr. 2016, whose complaint were acute vertigo or dizziness were retrospectively collected. All patients arrived at the emergency department within 24 hr of symptom onset, had no acute ischemic lesion first CT and NIHSS score of 0. Patients were divided into cerebral infarction group and noncerebral infarction group according to subsequent cerebral imaging results and clinical and laboratory factors related to cerebral infarction were analyzed.

**Result:**

51.6% of patients were female (*n* = 141). 46 patients (16.8%) were diagnosed with acute cerebral infarction. Baseline demographic data of the two groups was not significantly different. Univariate analysis found that history of smoking (*p* = 0.009), headache (*p* = 0.028), unsteadiness (*p* = 0.009), neuron specific enolase (*p* = 0.001), and vertebral artery abnormalities found on imaging (*p* = 0.009) were the significant difference between two groups. Increased neuron specific enolase (*p* = 0.005) and an abnormal vertebral artery (*p* = 0.044) were significant on multivariate analysis.

**Conclusions:**

16.8% of acute isolated vertigo or dizziness presentations were diagnosed with acute cerebral infarction. Increased serum neuron specific enolase and vertebral artery abnormalities were the strongest indicators of acute cerebral infarction.

## 1 INTRODUCTION

Acute onset vertigo or dizziness is a common symptom of posterior circulation ischemia. When vertigo or dizziness occurs in isolation or is accompanied by nausea and vomiting with a lack of other symptoms or signs of neurological impairment, the presentation is often presumed to be due to a benign peripheral cause, such as vestibular neuronitis (Hotson & Baloh, 1998). In recent years, cerebrovascular disease as a cause of isolated vertigo or dizziness has gained increased attention. Norrving, Magnusson, and Hlotás (1995) described 24 patients with acute isolated vertigo, age 50–75 years, in whom cerebellar infarction was found to be the cause in 25%. A retrospective analysis reviewed 907 case of dizziness visited in emergency department (ED) and found 37 cases (4%) were vascular origin. The vascular dizziness were associated with age >60 years old, accompanying imbalance and focal neurological deficits (Navi et al., 2012). Researches also suggested that vertebral artery hypoplasia, posterior circulation stenosis, and diabetes were correlated with stroke (Mosarrezai, Toghae, Majed, & Aloosh, 2012; Zhang et al., 2017). If a misdiagnosis occurs, patients may deteriorate resulting in permanent disability or a life‐threatening course. If such patients can be accurately diagnosed at an early stage and receive proper treatment, their outcomes may significantly improve. Therefore, we conducted a single‐center retrospective analysis of patients with isolated vertigo or dizziness to determine the factors related to a cerebrovascular etiology.

## 2 METHODS

### 2.1 Subjects

We retrospectively collected data of patients with isolated vertigo or dizziness who were hospitalized in the neurology department of East Hospital from January, 2015 to April, 2016.Vertigo and dizziness were defined in accordance with the International Classification of Vestibular Disorders (Bisdorff, Von Brevern, Lempert, & Newman‐Toker, 2009). “Vertigo” is the sensation of self‐motion when no self‐motion is occurring or the sensation of distorted self‐motion during an otherwise normal head movement. “Dizziness” is the sensation of disturbed or impaired spatial orientation without a false or distorted sense of motion. Patients' first neurological deficits were measured by a senior neurologist using the National Institutes of health Stroke Scale (NIHSS) in the ED. The study was approved by the Ethics Committee of East Hospital and the informed consent was obtained from the patients.

#### 2.1.1 Inclusion criteria

(a) Age ≥18 years; (b) Acute onset vertigo or dizziness accompanied by nausea, headache, and unsteadiness; (c) The interval between symptoms onset and first visit of ED was ≤24 hr;(d) The first NIHSS score was 0 as assessed by a neurologist; (e) The first cerebral CT showed no acute ischemic lesion and subsequent cerebral MRI or repeat CT confirming a diagnosis of acute cerebral infarction.

#### 2.1.2 Exclusion criteria

(a) First NIHSS score ≥ 1, including score for limb ataxia; (b) Dizziness caused by systemic diseases such as cardiac insufficiency, fever, and hypoglycemia; (c) The interval between onset of symptoms and first ED attendance>24 hr.

### 2.2 Data collection

We collected data including the patient's age, gender, interval between onset and first visit to the ED; past medical history, nature of dizziness ‐ persistent or paroxysmal, and associated symptoms, presence of nystagmus, first assessment of blood pressure; and first laboratory examination results including neuron specific enolase (NSE) within 48 hr of symptom onset. Serum NSE level is determined by electrochemiluminescence immunoassay and reported automatically, which can be done within 18 min.

### 2.3 Evaluation of Imaging

All patients underwent cerebral CT examination within 30 min of ED arrival. A cerebral MRI and MRA(Philips 1.5T, including T1WI, T2WI, DWI, and FLAIR sequence, if contraindicated, a repeat cerebral CT and CTA were conducted), neck vessel CTA (Toshiba, 320‐row)or MRA were conducted within 72 hr of symptom onset. Patients were divided into a cerebral infarction group and a noninfarction group according to cerebral MRI or repeat cerebral CT findings. Vertebral artery abnormalities were defined as follows:stenosis, narrow, distorted, or absent of unilateral vertebral artery in CTA/MRA image.

### 2.4 Statistical method

Statistical analysis was conducted with SPSS 20.0. Chi‐square analysis, *t*‐test and Mann–Whitney test were performed for univariate analysis. Cerebral infarction confirmed by MRI/CT was used as the dependent variable, factors with *p* < 0.05 on univariate analysis and gender, age were used as co‐variables to assess in a multivariate logistic regression model. A *p* < 0.05 was deemed statistically significant.

## 3 RESULTS

**Figure 1:**
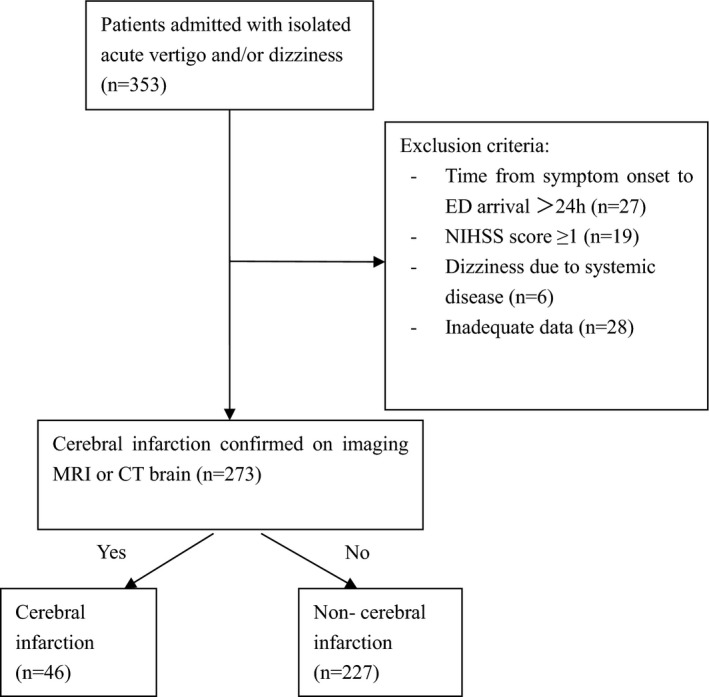
Study flow chart

During the study period, there were 353 patients hospitalized with vertigo or dizziness, 80 patients were excluded and 273 patients were included in the final analysis, details showed in study flow chart (Figure 1). The mean age was 67.1 ± 12.0 years and 141 cases were female (51.6%).46 patients were diagnosed with acute cerebral infarction through cerebral imaging (16.8%, 45 cases with MRI and 1 case with repeat CT).The smoking history was significantly higher in the cerebral infarction group (37% vs. 19.4%, *p* < 0.05). There was no statistically significant difference between the two groups regarding other baseline characteristics (Table 1).

**Table 1:** Patient characteristics, vascular risk factors, time to Emergency Department presentation, and blood pressure on arrival in cerebral infarction group and noncerebral infarction group presenting with acute vertigo or dizziness

Patient characteristics	Cerebral infarction *n* = 227	Non‐cerebral infarction *n* = 46	*p* value
Demographic			
Male	26 (57)	106 (47)	0.224
Age, Mean ± *SD* (years)	66.6 ± 12.5	66.2 ± 11.9	0.765
Vascular risk factors			
Hypertension	34 (74)	141 (62)	0.128
Diabetes mellitus	10 (22)	43 (19)	0.662
Atrial fibrillation	4 (9)	10 (4)	0.262
Coronary heart disease	5 (11)	23 (10)	0.881
Hyperlipemia	3 (7)	7 (4)	0.295
Smoking history	17 (37)	44 (19)	0.009^a^
Family history of stroke	0	3 (1)	0.291
Prior TIA/stroke	9 (20)	51 (23)	0.665
Cancer history	2 (4)	17 (8)	0.420
Time to ED presentation, hr, Median (IQR)	5 (8)	5 (13)	0.871
Initial systolic BP > 160 mmHg	13 (28)	45 (20)	0.202
Initial diastolic BP > 90 mmHg	10 (22)	50 (22)	0.966

Notes

Data are *n* (%) of total cohort unless otherwise specified. TIA indicates transient ischemic attack.

BP: blood pressure.

a Statistically significant, *p* < 0.05.

**Figure 2:**
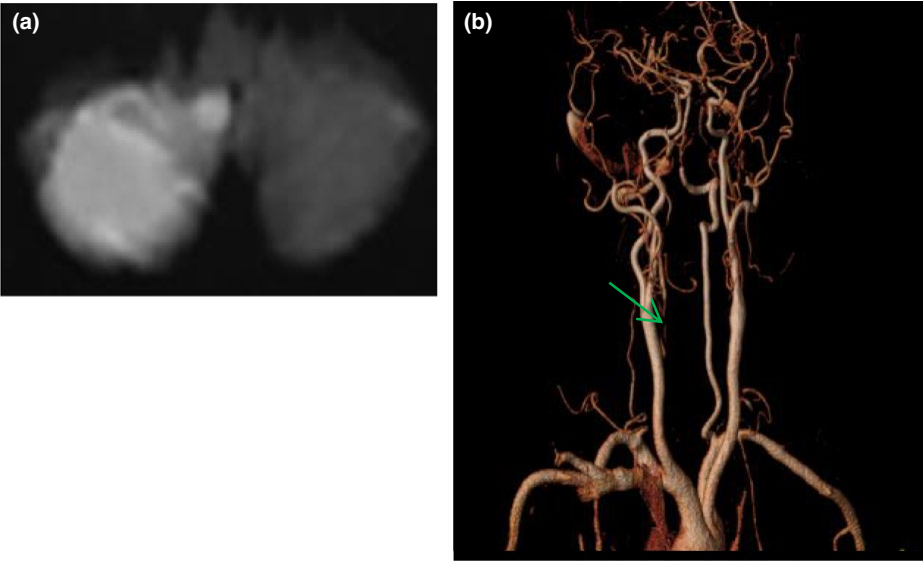
MR image of a 61‐year old man with acute vertigo. DWI showed acute infarction in right cerebellar hemisphere (a), neck vessel MRA showed proximal segment of right vertebral artery absent (green arrow, b)

**Figure 3:**
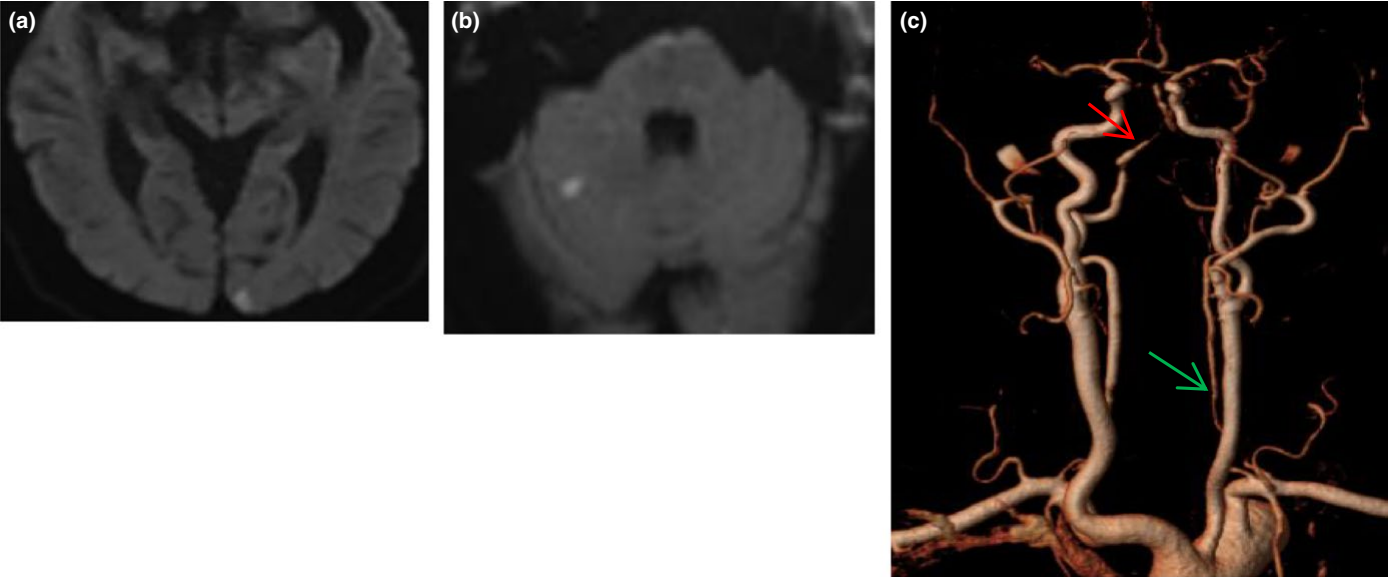
A 70‐year old man with acute vertigo and vomiting arrived ED 2.5 hr after onset and received MRI 28h after onset; his NIHSS was 0 during this time. Cerebral MRI showed left occipital (a) and right cerebellar hemisphere(b) infarction, left vertebral artery was narrow (green arrow) with right vertebral artery stenosis (red arrow, c)

**Figure 4:**
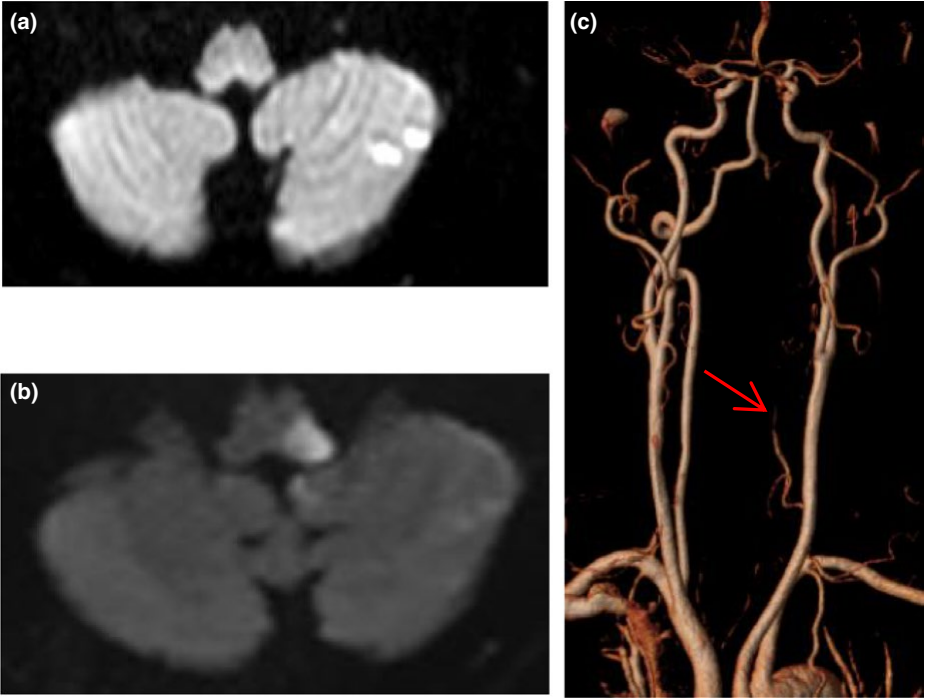
DWI image of a 61‐year old man with transient isolated vertigo showed left cerebellar infarction (a), his symptoms deteriorated 20h after onset and repeat MRI showed medullary infarction (b), the left vertebral artery was narrow and segmental absent (red arrow, c)

Among the 46 patients with acute cerebral infarction, seven patients progressed resulting NIHSS ≥1 within 24 hr. The average time from ED presentation to deterioration was 5 hr. 10 patients had 2 or more regions of acute infarction. The lesions involved the posterior circulation in 42 patients including the cerebellum (*n* = 25), thalamus (*n* = 9), occipital lobe (*n* = 8), and brainstem (*n* = 7). Four patients were anterior circulation territory infarction including the frontal lobe, corpus callosum, and centrum semiovale (Figures 2, 3, 4).

The frequency of headache when comparing cerebral infarction with noninfarction patients was 8.7% vs. 1.8% ( *p* < 0.05). The proportion of patients with symptoms of unsteadiness was also higher in the cerebral infarction group(37% vs. 19.4%, *p* < 0.05). Other accompanying symptoms and signs did not significantly differ between the two groups (*p* > 0.05) (Table 2).

**Table 2:** Symptoms and signs of the patients with acute vertigo or dizziness

Symptoms and signs	Cerebral infarction *n* = 227	Noncerebral infarction *n* = 46	*p* value
Vertigo, duration >1 hr	28 (61)	118 (52)	0.270
Rotational vertigo	34 (74)	148 (65)	0.253
Correlation with body posture	16 (35)	106 (47)	0.138
Nausea and vomiting	34 (74)	166 (73)	0.913
Tinnitus	3 (7)	21 (9)	0.537
Hearing impairment	1 (2)	8 (4)	0.623
Headache	4 (9)	4 (2)	0.02^a^
Unsteadiness	17 (37)	44 (19)	0.009^a^
Nystagmus	5 (11)	29 (13)	0.721

Notes

Data are *n* (%) of total cohort.

a Statistically significant, *p* < 0.05.

There was an increase of serum NSE (normal range 0–16.3 ng/ml, cut‐off value is 11.85 ng/ml calculated by using ROC curve) when comparing cerebral infarction with noninfarction patients (45.7% vs. 22.5%, *p* < 0.05). Other laboratory factors did not significantly differ (*p* > 0.05) (Table 3).

**Table 3:** Laboratory results in patients with cerebral infarction and noncerebral infarction presenting with isolated vertigo or dizziness

Laboratory examination	Cerebral infarction *n* = 227	Noncerebral infarction *n* = 46	*p* value
Leukocyte > 10*10^9^/L	10 (22)	29 (13)	0.113
Hb < 110 g/L, > 160 g/L	8 (17)	25 (11)	0.226
Platelets > 300*10^9^/L	4 (9)	17 (8)	0.783
D‐dimer > 0.55 mg/L,	14 (30)	49 (22)	0.194
Fibrinogen > 4 g/L	9 (20)	35 (15)	0.486
CRP >5 mg/L	12 (26)	65 (29)	0.726
Urinary protein(+)	18 (39)	58 (26)	0.061
Glucose > 6.1 mmol/L,	19 (41)	78 (35)	0.402
HbA1C > 6.0%	14 (30)	65 (29)	0.806
UA > 370umol/L	13 (28)	53 (23)	0.478
LDL > 2.6 mmol/L	33 (72)	149 (66)	0.424
Homocystine > 15.4umol/L	10 (22)	44 (19)	0.715
NSE > 11.85 ng/ml	21 (46)	51 (23)	0.001^a^

Notes

Data are *n* (%) of total cohort unless otherwise specified, CRP indicates C reactive protein.

Hb: hemoglobin; HbA1c,Glycated hemoglobin; LDL: low density lipoprotein; NSE: neuron specific enolase; UA: uric acid.

a Statistically significant, *p* < 0.05.

All patients underwent cerebral MRA or CTA, 130 patients underwent CTA or MRA examination of neck blood vessels. The abnormalities of vertebral artery in CTA/MRA image included: Segmental stenosis of the vertebral artery (*n* = 56), hypoplasia of unilateral vertebral artery (*n* = 58), and in four patients, a distorted vertebral artery was found. In 10 cases there was segmental absent of one vertebral artery. The occurrence rate of an abnormal vertebral artery when comparing the cerebral infarction group with the noninfarction group was 65.2% vs. 43.2% (*p* = 0.009).

Multivariate logistic regression analysis found that an increased NSE (OR 2.694, 95% CI 1. 346–5.392, *p* = 0.005) and an abnormality of the vertebral artery (OR 2.049, 95% CI 1.018–4.123, *p* = 0.044) were the strongest indicators of acute isolated vertigo of cerebrovascular origin (Table 4).

**Table 4:** Multivariate logistic regression analysis results in patients with cerebral infarction presenting with acute isolated vertigo or dizziness

Variate	OR (95% CI)	*p* value
Age	1.002 (0.973–1.033)	0.876
Gender	1.221 (0.508–2.935)	0.655
Smoking	0.39 (0.151–1.011)	0.053
Unsteadiness	0.56 (0.267–1.172)	0.124
Headache	0.241 (0.05–1156)	0.075
NSE > 11.85 ng/ml	2.694 (1.346–5.392)	0.005^a^
Abnormality of vertebral artery	2.049 (1.018–4.123)	0.044^a^

Notes

CI indicates confidence interval; NSE: neuron specific enolase; OR: odds ratio.

a Statistically significant, *p* < 0.05.

## 4 DISCUSSION

Isolated vertigo or dizziness is a common clinical symptom. A German study reported that 5% of adults experience symptoms of vertigo each year, of whom 80% consult a doctor (Neuhauser et al., 2005). A single‐center study found that 11% of ED visits related to complaints of vertigo or dizziness (Lammers et al., 2011). Isolated vertigo or dizziness can be symptoms preceding posterior circulation stroke or the only symptom of cerebral infarction, results of other studies found that among patients with isolated vertigo or dizziness, about 0.7%–10% are due to acute cerebral infarction (Doijiri, Uno, Miyashita, Ihara, & Nagatsuka, 2016; Kerber, Brown, Lisabeth, Smith, & Morgenstern, 2006; Mosarrezai et al., 2012; Navi et al., 2012; Paul, Simoni, & Rothwell, 2013). In the present study, we found a diagnosis of cerebral infarction among 46/273 (16.8%) hospitalized patients presenting acutely to ED with isolated vertigo or dizziness. The higher percentage found in our study may be due to differences in selection criteria.

Within our cohort, 91% of acute cerebral infarctions were located in the posterior circulation, most commonly the cerebellum. We also found four patients with infarction of the anterior circulation. It is postulated in these cases, that the lesions affected multisensory vestibular cortical networks involved in perceptive function, such as the thalamus, temporoparietal cortex, and peninsula adjacent to the inferior frontal gyrus (Dieterich & Brandt, 2008; Von Brevern, Süβmilch, & Zeise, 2014).

The initial NIHSS score of all cerebral infarction patients in the present study was 0.Symptoms such as dizziness, headache, and nausea, do not constitute points using the NIHSS (Martin‐Shild et al., 2011). These findings indicate that NIHSS scoring is insensitive and misleading if it is used to guide decision‐making for posterior circulation infarction. It is not uncommon for patients with acute vestibular symptoms due to stroke to be either initially misdiagnosed with peripheral vestibular disease or have a delay in definitive diagnosis (Calic, Cappelen‐Smith, Anderson, Xuan, & Cordato, 2016). Moreover, DWI is associated with false negative results when performed very early, especially for posterior circulation infarction (Zuo et al., 2015). For these reasons, identification of factors that may indicate which patients have isolated vertigo or dizziness caused by cerebral infarction is clinically important.

We found smoking history, headache, unsteadiness were different between cerebral infarction group and noncerebral infarction group. Other vascular risk factors including atrial fibrillation were not associated with acute cerebral infarction. Recent research showed patients with vertigo had a 3.01 times higher risk for stroke than the control group. And patients with more than three risk factors had a 5.51‐fold higher risk for stroke (Lee et al., 2011). The results of patients with vascular risk factors in this article were quite different from our study probably because of different concerns and inclusion criteria. Compared with the anterior circulation, posterior circulation infarction is more commonly accompanied with headache (Mitsias, Ramadan, Levine, Schultz, & Welch, 2006), which may be diffuse or localized to the occipital scalp (Kumral, Bogousslavsky, Van Melle, Regli, & Pierre, 1995). Other studies have found that the majority of patients with vertigo due to cerebellar infarction report accompanying imbalance (Lee et al., 2006). However, the smoking history, symptoms of headache and unsteadiness showed no significant association of cerebral infarction in regression analysis of our study.

We found an elevated NSE (>11.85 ng/ml)was associated with cerebral infarction in patients with acute vertigo or dizziness. NSE is located in cytoplasm of nerve cells or neuroendocrine cells, and it is released into blood and cerebrospinal fluid (CSF) when brain tissue is damaged (Isgrò, Bottoni, & Scatena, 2015). Animal experiments have verified that plasma NSE begins rising within 2 hr of ischemia, and continues for 2.5 days (Barone et al., 1993). Stevens, Jakobs, de Jager, Cunningham, and Korf (1999) observed that in 19 patients with acute cerebral embolism, serum NSE levels increased within 4 hr of the attack. Gruener, Gross, Gozlan, and Barak (1994) found that NSE levels in blood and CSF of patients with acute stroke reached peak values after 7 days. Increased levels of NSE have also been reported in a variety of other neurological disorders including Guillain‐Barré syndrome and cerebral trauma as well as specific neuroendocrine tumors (Isgrò et al., 2015). Our study findings suggest that an elevated NSE may be a useful indicator of a diagnosis of stroke in patients with acute isolated vertigo or dizziness.

Hypoplasia of the vertebral artery refers to an artery diameter of ≤2 mm. Hypoplasia and arteriosclerosis stenosis of the vertebral artery are commonly associated with ischemic stroke, especially accompanied with other risk factors (Katsanos, Kosmidou, & Kyritsis, 2013; Park, Kim, & Roh, 2007; Zhang et al., 2017). In our study, 65.2% of patients with acute vertigo or dizziness due to cerebral infarction had vertebral artery abnormalities, which was significantly higher than that found in the noninfarction group.

There is literature showing that an increase of blood leukocytes is related to severity of cerebral infarction (Kim et al., 2012) and if vertigo patients have accompanying systolic blood pressures >160 mmHg, it is more likely to be of central origin (Okada, Nakagawa, & Inokuchi, 2012). In this study, univariate analysis for the patients' white blood cell counts and first systolic blood pressures were not associated with cerebral infarction. The lack of significance in our study may relate to patient inclusion criteria.

Finally, the head impulse‐nystagmus‐test of skew (HINTS) examination is a bedside method used to differentiate peripheral from central vertigo. The presence of a normal horizontal head impulse test, direction‐changing nystagmus or skew deviation were highly indicative of a stroke with 100% sensitivity and 96% specificity (Kattah, Talkad, Wang, Hsieh, & Newman‐Toker, 2009).However, Kim, Park, Kim, and Kim (2015) proposed that positive results from a head impulse test may occur in central vestibular lesions. Among patients in the present study, more than half reported persistent vertigo, so it was difficult completing a bed‐side HINT assessment in ED for most of these subjects. Future use of video‐assisted vestibular assessment in the ED may improve the diagnostic accuracy of HINTS examination in such cases.

Limitations of the present study included its retrospective single‐center design and analysis only of patients hospitalized for isolated vertigo or dizziness. Future, prospective studies with a large sample size are warranted to confirm our findings.

## 5 CONCLUSION

In this study, 16.8% of patients hospitalized with acute isolated vertigo or dizziness had underlying acute cerebral infarction. An increased NSE and abnormal CTA/MRA imaging of the vertebral artery may be markers of acute cerebral infarction in patients presenting to the ED with acute isolated vertigo or dizziness. Prospective studies are warranted to evaluate whether NSE is an early biomarker of cerebral infarction in acute vertigo or dizziness.

## CONFLICT OF INTERESTS

The authors declare no financial or other conflicts of interest.
